# Students’ Entire Deep Learning Personality Model and Perceived Teachers’ Emotional Support

**DOI:** 10.3389/fpsyg.2021.793548

**Published:** 2022-01-13

**Authors:** Enyun Liu, Jingxian Zhao, Noorzareith Sofeia

**Affiliations:** ^1^Shandong Women’s University, Jinan, China; ^2^Faculty of Education, Languages and Psychology, SEGi University, Kota Damansara, Malaysia; ^3^Center for Teaching and Learning, SEGi University, Kota Damansara, Malaysia

**Keywords:** deep learning, deep learning investment, deep cognitive-emotional experience, deep information processing, deep learning meta-cognitive, perceived teacher emotional support

## Abstract

In recent years, deep learning as the requirement of higher education for students has attracted the attention of many scholars, and previous studies focused on defining deep learning as the deep processing of knowledge of the brain, however, in the process of knowledge processing, the brain not only involves the deep processing of information but also participates in learning consciously and emotionally. Therefore, this research proposed a four-factor model hypothesis for deep learning that includes deep learning investment, deep cognitive-emotional experience, deep information processing, and deep learning meta-cognitive. In addition, the research proposed teachers’ emotional support perceived by students has an effect on the four factors of deep learning. Through SPSS 26 and AMOS 24, this research has verified the four-factor model of deep learning applying exploratory factor analysis (EFA) and confirmatory factor analysis (CFA) and verified that the perceived teacher emotional support has an impact on the four factors of students’ deep learning using the SEM.

## Introduction

In recent years, deep learning has attracted great attention in the field of higher education (Chotitham et al., 2014), and high imagination and creativity, emphasizing cooperation, coordination, integration, interpretation, personalization, and learning to learn will be the major requirement for individuals to satisfy the needs of talents of social development (Esteban-Guitart and Gee, 2020), thus higher education should cultivate students to satisfy the requirement of social development. Deep learning emphasizes the in-depth participation of students in the learning process, broader understanding of knowledge, achieving intrinsic interest and ability, seeking meaning between content, connecting ideas with previous knowledge and daily experience, understanding all presenting materials, participating in the content of the course, collaborating with others, using evidence to test logic, and the various advanced abilities which meet the requirements of higher education for the development of students’ ability (Asikainen, 2014; Faranda et al., 2020).

Previous studies focused on defining deep learning as the deep processing of knowledge by the brain, and related research was conducted on this basis (Marton and Säljö, 1976; Entwistle, 1981; Biggs, 1987, 1989, 2003; Biggs et al., 2001; Ramsden, 2003; Tagg, 2003). However, in the process of knowledge processing, the brain not only involves the deep processing of information but also participates in the process of deep learning consciously and emotionally (Tallis and Aleksander, 2008; Xie et al., 2016). Furthermore, Karagiannopoulou and Entwistle (2019) also believed that deep learners pursue broader and deeper academic understanding and ideological exchange, and their emotional and conscious needs in the learning process are more obvious, which also showed that deep learning not only includes the process of deep information processing but also involves emotional and conscious participation. However, in the previous research, there was no theoretical model of deep learning containing information processing, emotion, and consciousness. Therefore, this study explored students’ deep learning from the perspective of overall personality development.

According to cognitive affective personality system theory (CAPS), when students are in the state of deep cognitive process, the cognitive personality system of students will interact with their emotional state which will affect the cognitive emotion or behavior results (Mischel and Shoda, 1995). This theory illustrated that students’ personal experience and emotional state interact in the process of deep learning and produce different deep learning results. Postareff et al. (2017) also showed that the emotions experienced in the learning process, such as “enjoyment,” “relaxation,” “boredom,” and “anxiety,” are all related to the learning results. In addition, teachers’ positive emotions such as guidance, understanding, help, and support can also affect students’ learning state and emotional state (Karagiannopoulou and Entwistle, 2019). Therefore, based on CAPS theory, the current research assumed that deep learning includes emotional factors, and deep learning is also influenced by perceived teachers’ emotional support.

### The Deep Learning Four-Factor Model

The concept of deep learning was proposed by Marton and Säljö (1976) who used phenomenological research methods to classify students’ reading styles and divided them into deep learning methods and shallow learning methods. Students who adopted deep learning methods have learning intention of extracting meaning from an article and connect with one’s previous knowledge and their learning strategy is to combine the thoughts into a whole structure, critically evaluate the knowledge and conclusions in the article. On the other hand, the learning intention of the shallow learning method is memory, and shallow learning strategy is mechanical processing (Marton and Säljö, 1976). Biggs (1987) proposed that the strategic characteristics of deep learning include interest in learning topics and willingness to experience and participate in the learning process of a certain topic, looking for the inner meaning and connection between learning content, and combining the new knowledge with original knowledge in the cognitive structure, combining with the real world for induction and deduction, and meaningful learning content is encoded and stored in the long-term memory of the mind for application. According to Bloom’s cognitive target classification, the cognitive level of shallow learning only stops at knowing knowledge and comprehensive, while the cognitive level of deep learning includes application, analysis, synthesis, and evaluation (Laird et al., 2008; Xie et al., 2016). Hattie and Donoghue (2016) proposed that deep learning refers to finding meaning, connecting and expanding ideas, and finding patterns and underlying principles. Deep learning also includes understanding and using the relationship between concepts and program knowledge through the ability to apply conceptual knowledge in new contexts (Hattie and Donoghue, 2016; Winch, 2017). Fullan et al. (2018) believed that deep learning is embodied in a strong sense of identity around goals or passions, creativity and mastery associated with valuable pursuits, as well as connections with the world and others.

Overall, the connotation of deep learning is constantly enriched. However, the keywords of deep learning focus on the levels of learning intention, learning strategy, meaningful learning, long-term memory, and application. Fullan et al. (2018) put forward that deep learning not only includes the level of deep cognition and application of knowledge, but also includes identity and connection with the world, and then turned to a more comprehensive study of deep learning. According to previous research, deep learning researches mainly focused on deep understanding and information processing of knowledge content when students are learning and did not pay attention to the emotional state of students. However, learning is a comprehensive process, when students learn deeply, it not only involves the processing of knowledge but also involves the influence of many factors such as emotion, interest, enthusiasm, appreciation, evaluation, and identification (Esteban-Guitart and Gee, 2020). However, at present, there is no research to analyze the connotation of deep learning from the emotional aspect. Therefore, the current research proposed a four-factor model of deep learning that includes cognitive-emotional factors and investigated the effectiveness of the four-factor model of deep learning based on the structural equation model.

In previous studies, Biggs (1987) proposed that deep learning is to link knowledge, combine new knowledge into the original knowledge structure, form a long-term memory of knowledge, and be able to summarize and deduce knowledge to achieve meaningful learning. Based on this, the current research proposed the first dimension of deep learning, that is, deep information processing, which represents a deep understanding of knowledge and a meaningful construction with the original learning experience.

In addition, Biggs (1987) put forward that deep learning strategies are students’ active participation and investment in the learning process of a certain topic, which indicates that students’ active participation in the learning process in deep learning state rather than passive learning or passive investment. Therefore, the current research put forward the second dimension of deep learning, that is, deep learning investment, which represents students’ active participation and investment in the process of deep learning. Therefore, based on the deep learning definition of Biggs (1987), the current research put forward the dimension of deep information processing and the dimension of deep learning investment.

There is no researcher who theoretically verified whether deep learning includes cognitive emotion. Therefore, according to the CAPS theory, students’ emotions will also participate in the cognitive process and influence learning behavior or results (Mischel and Shoda, 1995), thus in the process of deep learning, cognitive emotion will also participate in the process of deep learning and influence the learning state of individuals. Therefore, the current research put forward the dimension of cognitive emotion experience, which represents the emotional state that individuals have when students are in the deep learning state.

Based on putting forward the dimension of deep information processing, deep learning investment, and deep cognitive-emotional experience, according to the self-regulatory executive function (S-REF) model, meta-cognitive factors involved in self-regulation in the entire process of cognition, emotion, and behavioral beliefs (Wells and Matthews, 1996; Wells, 2009), Cartwright-Hatton et al. (2004) put forward that the beliefs of meta-cognitive participation include promotes reflection, and this kind of reflection can lead to circular thinking patterns, overcome emotional difficulties, keep self-attention, control thoughts, that is to say, the meta-cognitive process participates in the deep learning information processing process, cognitive emotion experience process and investment process, and adjusts these beliefs or states to maintain deep learning beliefs. Therefore, the current research proposed the meta-cognitive dimension of deep learning.

Therefore, this research put forward hypothesis 1: Deep learning four-factor model includes deep information processing, deep learning investment, deep cognitive emotional experience, and deep learning meta-cognitive.

### The Relation Between Perceived Teacher Emotional Support and Deep Learning

Perceived teacher emotional support usually refers to students’ perceptions of enthusiasm, friendliness, and care for teachers (Ryan and Patrick, 2001; Federici and Skaalvik, 2013), and includes three dimensions, which are positive atmosphere which is the teacher’s ability to create a positive interaction atmosphere with students, teacher sensitivity which is to what extent teachers are willing to respond to students’ academic and emotional needs, and emphasis on student personality which is to what extent teachers provide students with autonomy and focus on the development of students’ overall personality (Pianta and Hamre, 2009). According to CAPS theory (Mischel and Shoda, 1995), the individual’s self-personality system will have emotional reactions in the cognitive process. Moreover, under different emotional support environments, the emotional reactions of individuals will be different, and the environment with more emotional support will help individuals’ cognitive and emotional development (Atoum and Al-Shoboul, 2018; Karagiannopoulou and Entwistle, 2019; Romano et al., 2020, 2021). In the teaching process, teachers are the main body to provide students with emotional support factors (Ryan and Patrick, 2001). Students need a certain degree of attention and support from teachers and the relationship between teachers and students can influence students’ status and ability development (Ryan and Patrick, 2001; Burić, 2019), and the use of deep learning methods are closely related to students’ perception of the learning environment (Nijhuis et al., 2005; Biggs and Tang, 2007). Studies have also shown that when teachers show students’ autonomy, supportive and emotional caring teaching behaviors, they will have an impact on students’ learning participation and emotions (Reeve and Jang, 2006; Karagiannopoulou and Entwistle, 2019), and when students are in a learning environment that can provide students with more opportunities to master knowledge independently, help students reflect, and actively construct a knowledge framework, deep learning is more likely to occur (Warburton, 2003). Studies have shown that teacher emotional support is significantly correlated with students’ learning motivation, emotional and behavioral outcome investment (Sakiz et al., 2012). Stipek et al. (1998) found that the teacher’s characteristics of listening, respect, recognition, and fair treatment of students’ emotional support for students in the classroom will affect the degree of participation of students and the degree of active learning of students in the classroom (Murdock, 1999; Becker and Luthar, 2002). All these studies demonstrated that teachers’ emotions are related to students’ learning to some extent.

In addition, many studies have shown that an effective teaching environment should promote students’ deep learning, and the methods to promote students’ deep learning should focus on students’ intrinsic emotional support needs, take students as the center, encourage students to explore teaching methods independently, and allow students to generate learning interest during the learning process (Entwistle and Ramsden, 1983; Biggs, 1988, 1989; Whelan, 1988; Prosser and Millar, 1989; Ramsden, 2003; Tagg, 2003; de la Fuente, 2021). Studies have also shown that deep emotional investment must precede investment in learning behavior to detect whether the environment is threatening to learn (Roeser et al., 1998; Christenson et al., 2012). When a balance between personal needs and the opportunities, goals, and values provided by the environment is achieved, it can make people respond to the surrounding environment mentally, emotionally, and behaviorally (Caplan and Van Harrison, 1993). Therefore, the support of emotional factors is very important for students’ deep learning, and teachers, as the main body providing emotional support for students in the teaching and learning environment, play an indispensable role.

However, there are no articles that explored the relationship between perceived teacher emotional support and deep learning. Therefore, this research proposed hypothesis 2: Perceived teachers’ emotional support can significantly predict deep learning four factors.

### Research Aims

#### Aim 1

To explore deep learning from a more comprehensive perspective which includes deep information processing, deep learning investment, deep cognitive emotional experience, and deep learning meta-cognitive. Additionally, to explore the major implication of these four factors which lies in forming students’ entire deep learning personality model, representing a more comprehensive deep learning theoretical model.

#### Aim 2

Students’ deep learning emotional state not only includes inner emotional experience but is also influenced by external emotional factors, and teachers as the main external environment factor, and this study will make a verification whether students perceived from their emotional support will affect on students’ deep learning state.

### Research Hypotheses

#### Hypothesis 1

Deep learning four-factor model includes deep information processing; deep learning investment; deep cognitive emotional experience; and deep learning meta-cognitive.

#### Hypothesis 2

Perceived teachers’ emotional support can significantly predict deep learning four factors.

## Method

Exploratory factor analysis (EFA) and confirmatory factor analysis (CFA) are increasingly used to measure in the education field (Istiyono, 2019; Harerimana and Mtshali, 2020). In this study, EFA and CFA are used to verify the four-factor model of deep learning, to verify that emotional factors can be one of the factors of deep learning, and to verify the effect of perceived teacher emotional support on students deep learning by using the structural equation model.

### Participants

The main survey object of this research is the students at L University, Shandong Province, China, a local comprehensive university. Through stratified random sampling, students from four grades, 23 institutes, and 36 majors were selected as samples, 240 freshmen, 266 sophomores, 232 juniors, and 190 seniors, accounting for 26, 28.4, 25.1, and 20.5% respectively; liberal arts students (250), science students (149), engineering students (288), and arts and physical students (241), accounting for 26.9, 16, 31, and 25.9% respectively; 387 boys and 541 girls, accounting for 37.5 and 62.5%, respectively. The sample distributions were relatively balanced. A total of 928 questionnaires were issued in this survey, and a total of 865 valid questionnaires were returned. The effective response rate was approximately 93.2%. Therefore, this data analysis result has representative.

### Measures

#### Deep Learning Four-Factor Questionnaires

This research was based on the hypotheses of the deep learning four-factor model including deep learning investment, deep cognitive-emotional experience, deep information processing, and deep learning meta-cognitive.

The sub-scale of the deep information processing whose aim is to measure the deep processing of knowledge and refers to students’ intelligence level, used the items in the Multiple intelligence profiling questionnaires (Tirri and Nokelainen, 2011) and revised them based on the actual conditions of local students to measure the level of students’ deep information processing. This sub-scale includes 4 Items (for example, I can use concept maps or mind maps to organize the knowledge I have learned and describe in my language), according to the Likert scale of 5 points to score, from 1 point (strongly disagree) to 5 points (strongly agree).

The sub-scale of the deep learning investment based on the Australasian Survey of Student Engagement questionnaire (Coates, 2010) and revised according to the actual situation of local students to measure the level of students’ deep learning investment, mainly focusing on deep learning investment level, this sub-scale includes 8 items (for example, I will make full use of the library or online network resources to actively communicate with others about the knowledge I have learned after class), which is scored according to the Likert scale of 5 points, from 1 point (strongly disagree) to 5 points (strongly agree).

The sub-scale of the deep learning meta-cognitive based on the Meta-cognition Questionnaire-30 (Spada et al., 2008) and revised according to the actual situation of local students, to measure the level of students’ deep learning meta-cognition, namely for students’ deep learning belief levels, this sub-scale includes 4 items (for example, when I encounter difficulties or problems, I first consider what method to solve, and can always use this method to solve such problems), according to the Likert scale of 5 points, from 1 point (strongly disagree) to 5 points (strongly agree).

The sub-scale of the deep emotional experience items of students’ deep learning, including students’ sense of self-achievement, sense of insight, and sense of interest in learning which represents deep cognitive-emotional experience level. There were no suitable items to measure deep learning cognitive-emotional experience from other questionnaires, thus forming this scale from three aspects and containing 3 items (for example, I think I’m learning some academic topics are sometimes as exciting as reading a good novel or watching a good movie), according to the Likert scale of 5 points, from 1 point (strongly disagree) to 5 points (strongly agree).

#### Perceived Teacher Emotional Support Questionnaire

This research is based on the three dimensions of teacher emotional support: positive atmosphere which is the teacher’s ability to create an atmosphere for active interaction with students, teacher sensitivity which is how much teachers are willing to respond to students’ academic and emotional needs, and the impact on students’ personality value which is to what extent teachers provide students with autonomy and focus on the development of students’ overall personality (Pianta and Hamre, 2009) to form a teacher’s emotional support scale perceived by students. There were no suitable items to measure perceived teachers’ emotional support from other questionnaires, thus forming this scale from three aspects based on the definition of perceived teacher emotional support and containing 3 items (for example, in the teaching process, my teacher usually encourages students to participate in the teaching to make the teaching more lively), according to the Likert scale of 5 points to score, from 1 (strongly disagree) to 5 (strongly agree).

#### Procedure

This article contacted local schools and various institutions and asked for permission, explaining the goals and specific details of the research. Before doing research, tell students that their participation is voluntary and confidential to avoid possible adverse effects on students, and ask students to complete the questionnaire as truthfully as possible.

### Data Analysis

Perform descriptive analysis and Pearson’s correlation test to observe the correlation between variables. In order to verify the reliability and validity of the four-factor deep learning questionnaire, this study used SPSS 26 and AMOS 24 (International Business Machines Corporation, Armonk, New York, United States) analysis software to conduct EFA and CFA to verify questionnaires’ reliability and validity using different samples, thus verifying hypothesis 1, and then using structural equation model method to verify hypothesis 2.

## Results

### Preliminary Analysis

Descriptive statistics (mean and *SD*) of the four factors of deep learning and the perceived teacher emotion scale, Pearson’s correlation analysis and reliability test are shown in Table 1. The Cronbach’s alpha coefficients of the sub-scales were all greater than 0.60, indicating that questionnaires have good reliability. Deep learning investment and deep information processing (*r* = 0.6, *p* < 0.01), deep learning meta-cognition (*r* = 0.57, *p* < 0.01) and deep cognitive emotional experience (*r* = 0.46, *p* < 0.01) is positively correlated. Deep information processing is positively correlated with deep learning meta-cognition (*r* = 0.48, *p* < 0.01) and deep cognitive emotional experience (*r* = 0.4, *p* < 0.01). Deep learning meta-cognition is positively correlated with deep cognitive emotional experience (*r* = 0.41, *p* < 0.01). In addition, the perceived teacher emotional support and deep learning investment (*r* = 0.32, *p* < 0.01), deep information processing (*r* = 0.36, *p* < 0.01), deep learning meta-cognition (*r* = 0.33, *p* < 0.01) and deep cognitive emotional experience (*r* = 0.3, *p* < 0.01) are positively correlated. Therefore, the four factors of deep learning have significant internal correlations, and these four factors have significant correlations with perceived teacher emotional support.

**TABLE 1:** Descriptive statistics and correlation among variables.

Variables	Cronbach’s alpha	M	SD	DLI	DIP	DLMC	DCEE	PTES
Q1	0.816	3.19	0.65	1				
Q2	0.713	3.45	0.67	0.60**	1			
Q3	0.651	3.50	0.69	0.57**	0.48**	1		
Q4	0.603	3.47	0.79	0.46**	0.40**	0.41**	1	
Q5	0.740	3.88	0.72	0.32**	0.36**	0.33**	0.30**	1

N = 865. DLI, deep learning investment; DIP, deep information processing; DLMC, deep learning meta-cognitive; DCEE, deep cognitive emotional experience; PTES, perceived teacher emotional support. *p < 0.05, **p < 0.01.

### Exploratory Factor Analysis and Confirmatory Factor Analysis

**FIGURE 1:**
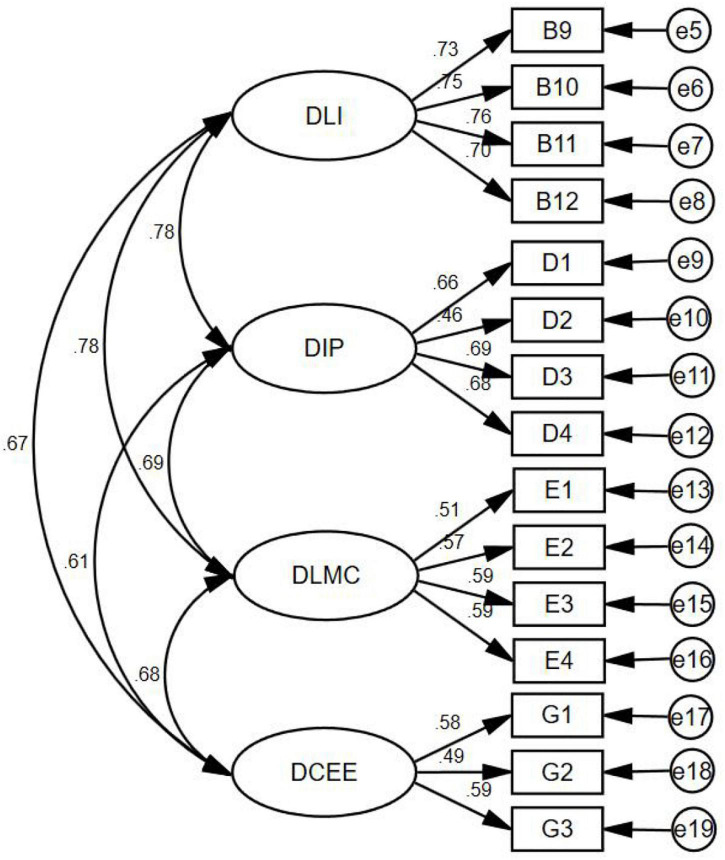
Confirmation factor analysis (DLI, deep learning investment; DIP, deep information processing; DLMC, deep learning meta-cognitive; DCEE, deep cognitive emotional experience; PTES, perceived teacher emotional support).

In the initial factor analysis phase (EFA), data were screened using the Kaiser-Meyer-Olkin (KMO) measuring of sampling adequacy (> 0.5) and Bartlett’s Test of Sphericity (< 0.05) (Tabachnick and Fidell, 2001; Taherdoost et al., 2014). Using principal component analysis to determine the number of factors that need to be retained in the model (Wood et al., 2015). According to Tables 2–5, and Figure 1, the analysis results show that KMO (0.919 > 0.5), Bartlett’s Test of Sphericity (*P* < 0.001), total variance explained > 50%, rotate to get four factors, and the initially items correspond to the CFA confirmatory model fit. The fit of the best model is critical to the model (Hooper et al., 2008). This study used model fit statistics including chi-square (233.283), probability level (0.000), chi-square/degrees of freedom (CMIN/*Df*) (2.777 < 3), comparative fit index (CFI) (0.959), incremental fit index (IFI) (0.959), TLI (0.948), root-mean-square error of approximation (RMSEA) (0.045 < 0.05), and standardized root mean square residual (SRMR) (0.0324 < 0.05) (MacCallum et al., 1996; Hu and Bentler, 1999; Hooper et al., 2008; Kline, 2011), which all indicated model fit good. Therefore, this result illustrated that the deep learning four-factor model was established, and hypothesis 1 was verified.

**TABLE 2:** KMO and Bartlett’s test.

Kaiser-Meyer-Olkin measure of sampling adequacy.		0.919
Bartlett’s Test of Sphericity	Approx. Chi-Square	4354.381
	df	171
	Sig.	0.000

**TABLE 3:** Total variance explained.

Component	Initial Eigenvalues			Extraction sums of squared loadings			Rotation sums of squared loadings		
	Total	% of variance	Cumulative %	Total	% of variance	Cumulative %	Total	% of variance	Cumulative %
1	6.07	31.96	31.96	6.07	31.96	31.96	3.11	16.35	16.35
2	1.26	6.61	38.57	1.26	6.61	38.57	2.44	12.85	29.20
3	1.10	5.79	44.36	1.10	5.79	44.36	2.22	11.70	40.89
4	1.07	5.62	49.98	1.07	5.62	49.98	1.73	9.08	49.98
5	0.90	4.73	54.71						

*Extraction method: Principal component analysis.*

**TABLE 4:** Rotated component matrix^a^.

	Component			
	1	2	3	4
B91	0.705			
B92	0.670			
B101	0.664			
B102	0.636			
B111	0.629			
B112	0.555			
B121	0.549			
B122	0.434			
D1		0.613		
D2		0.611		
D3		0.605		
D4		0.522		
E1			0.765	
E2			0.667	
E3			0.645	
E4			0.582	
G1				0.731
G2				0.721
G3				0.527

*Extraction method: Principal component analysis.*

*Rotation method: Varimax with Kaiser normalization.*

*^a^Rotation converged in six iterations.*

**TABLE 5:** Component transformation matrix.

Component	1	2	3	4
1	0.626	0.514	0.478	0.338
2	–0.642	0.412	–0.058	0.644
3	–0.053	–0.720	0.522	0.454
4	0.439	–0.219	–0.703	0.514

*Extraction method: Principal component analysis.*

*Rotation method: Varimax with Kaiser normalization.*

### Structural Equation Modeling

**FIGURE 2:**
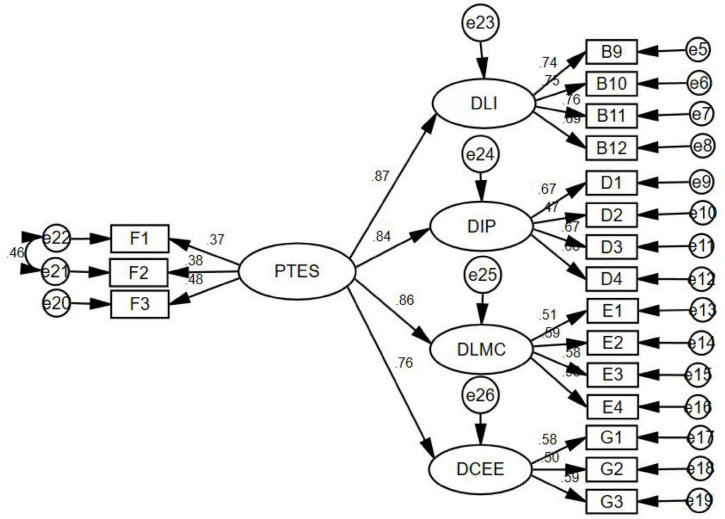
Structure equation model (DLI, deep learning investment; DIP, deep information processing; DLMC, deep learning meta-cognitive; DCEE, deep cognitive emotional experience; PTES, perceived teacher emotional support).

To evaluate the hypothetical model, this study uses model fit statistics including chi-square (510.035), probability level (0.000), CMIN/*Df* (3.923 < 3), CFI (0.914), IFI (0.914), TLI (0.899), RMSEA (0.058 < 0.08), and SRMR (0.0478 < 0.05) (MacCallum et al., 1996; Hu and Bentler, 1999; Hooper et al., 2008; Kline, 2011), which all indicated model fit good which demonstrated that the hypothesis model is established, specifically, perceived teacher emotional support can predict deep learning investment (β = 0.84, *p* < 0.001), deep information processing (β = 0.83, *p* < 0.001), deep learning meta-cognition (β = 0.85, *p* < 0.001), deep cognitive emotional experience (β = 0.76, *p* < 0.001) (shown in Figure 2). Therefore, it verified hypothesis 2, which asserted that perceived teachers emotional support can significantly predict deep learning four factors.

## Discussion

### The Deep Learning Four-Factor Model

This study verified the four-factor model of deep learning. Firstly, this study verified that deep learning includes two dimensions, deep information processing, and deep learning investment which proved the rationality of Biggs (1987) definition of deep learning from the model cognitive perspective, and then, this study also proved that deep learning not only includes deep information processing and deep learning investment but also contains the deep cognitive emotional experience and deep learning meta-cognitive participation, thus this study results supported the CAPS theory (Mischel and Shoda, 1995) that emotional factors of students will have an interaction with individuals’ cognitive and behavior results to form the individual’s inherent learning quality characteristics. Previous similarly studies also supported the current research results, Chong et al. (2018) proved that student emotional participation has a significant mediating effect on academic performance, and there is a symbiotic and coordinated relationship between learning emotion and mindful learning (Yanko and Yap, 2020), emotional factors play a significant mediating role between active learning and learning effectiveness (Kustyarini, 2020), all of these studies demonstrated that emotional factors participate in the learning process, therefore the current research conclusions are reasonable, and there is no research to verify whether the deep learning model contains emotional factors from the perspective of theoretical construction, so the current research conclusions are innovative.

Furthermore, the research results also supported the self-regulatory executive function (S-REF) model (Lewin, 1935; Murray, 1938, 1951) that meta-cognitive factor can be used as an important part of the deep learning model which participating in the entire process of students’ deep learning investment, deep information processing, and deep cognitive emotional experience to maintain students’ deep learning state. Previous similarly studies also supported the current research results, meta-cognitive adjustment is to ensure that students actively participate in learning activities and improve their understanding of learning content, highly motivated learning and learning plan, supervise and evaluate the learning process and manage thought activities (Bakar and Ismail, 2020; Hayat et al., 2020). Previous studies have shown that meta-cognition can adjust learning beliefs or learning states, but no research has been done to verify whether deep learning contains meta-cognition from the perspective of theoretical model verification. This current research conclusion proved the rationality of deep learning containing meta-cognition. The main purpose of current research is to explore whether deep learning contains cognitive-emotional factors, while, meta-cognition factors, as auxiliary factors of information processing, deep learning investment, and deep cognitive experience in deep learning, are generated based on other three factors.

### Perceived Emotional Support and Deep Learning

This study verified that perceived teachers’ emotional support has a significant influence on the four factors of deep learning, indicating that the emotional factors of students’ learning experience can be used as predictors of deep learning, and the emotional support of teachers to students will affect the overall state of deep learning which also support the CAPS theory (Mischel and Shoda, 1995) which means personal cognitive emotional will have an interaction between people emotion and the environment emotional support, and the deep learning of different individuals’ personality will interact with the perceived emotional support of teachers, forming an interactive system. Previous similarly studies also supported the current research results, a person’s physical or psychological needs, values, goals, abilities or personality needs and environment including internal and external rewards, roles, cultural values, etc. which all will have an interact with each other (French et al., 1982; Muchinsky and Monahan, 1987; Dawis, 1992; Kristof-Brown et al., 2005), and the learning style adopted by students is affected by factors in the learning environment (Gijbels et al., 2014), and teaching context plays a vital role in student learning achievement and learning experience process (Biggs, 1987; Eley, 1992; Biggs and Moore, 1993; Gow et al., 1994; Beatie et al., 1997; Zeegers, 2001; Tagg, 2003), and if teachers design the learning environment according to the emotional characteristics, the students will maintain higher contextual interest (Endres et al., 2020), and when students perceive that teachers have higher expectations for them, it will have a significant impact on students’ learning enthusiasm, and the different support they perceive will also affect students’ expectations for self-behavior (Weinstein and McKown, 1998; Danielsen et al., 2010), these studies have explained the correlation between teachers’ emotional support and students’ learning from different angles, and proved the rationality of the current research conclusions. Therefore, it is reasonable to conclude that the perceived emotional support of teachers has a significant predictive effect on the four factors of students’ deep learning.

In addition, this result has certain practical significance, when students perceived more emotional support from teachers, students will improve their deep information processing, deep learning meta-cognitive, deep cognitive emotional experience, and deep learning investment. Therefore, teachers should create a teaching environment with more emotional support to improve students’ deep learning and provide a more positive atmosphere for teaching, improve feedback on students’ learning needs and emotional needs, and focus on supporting students’ whole personality development to achieve supporting for students’ deep learning entire personality development (Pianta and Hamre, 2009; Karagiannopoulou and Entwistle, 2019; Romano et al., 2020, 2021; de la Fuente et al., 2021a,b) in the practical teaching and learning environment to improve students’ deep learning.

## Conclusion

In summary, this study explored deep learning connotation from a more comprehensive perspective, and the results verified deep learning model containing deep information processing, deep learning investment, deep cognitive-emotional experience, and deep learning meta-cognitive these four factors, indicating that the inner cognitive emotional experience of students during learning has an interaction with the overall state of students’ deep learning, and then, deep learning is also affected by the emotional support provided by external teachers. This research results filled in the gap of the deep learning theoretical model from an emotional perspective and the relationship between perceived teachers’ emotional support and deep learning. moreover, this research provided practical implications that in the practical teaching process, teachers should give more attention to students’ emotional needs to improve their deep learning entire personality development.

### Ethical Approval

Written informed consent to participate in this study was provided by the participants. All procedures performed in studies involving human participants were in accordance with the ethical standards of the institutional and/or national research committee and with the 1964 Declaration of Helsinki and its later amendments or comparable ethical standards. All research procedures have been approved by the current research institute in the L University, and the research procedures take full ethical issues into consideration.

### Limitations

Although this research has verified the deep learning four-factor model and found the relationship between perceived teacher emotional support and students’ deep learning, further research is needed on which factors will affect teacher emotional support to improve emotional support from teachers and then improve students’ deep learning. Therefore, it is necessary to conduct further qualitative and quantitative analysis. In addition, from the perspective of external validity, the research object of this study is undergraduates, so the conclusion cannot be extended to the general population. Moreover, the cross-sectional data do not allow causal inferences, so future research should conduct longitudinal research on the fundamental of the current results. Finally, this research data was collected from one university which may cause the result over-generation, thus more diversified data should be collected in further research to make the results more representative and generalized.

## Data Availability Statement

The original contributions presented in the study are included in the article/supplementary material, further inquiries can be directed to the corresponding author.

## Ethics Statement

Ethical review and approval was not required for the study on human participants in accordance with the local legislation and institutional requirements. Participants provided their written informed consent to participate in this study.

## Author Contributions

All authors listed have made a substantial, direct, and intellectual contribution to the work, and approved it for publication.

## Conflict of Interest

The authors declare that the research was conducted in the absence of any commercial or financial relationships that could be construed as a potential conflict of interest.

## Publisher’s Note

All claims expressed in this article are solely those of the authors and do not necessarily represent those of their affiliated organizations, or those of the publisher, the editors and the reviewers. Any product that may be evaluated in this article, or claim that may be made by its manufacturer, is not guaranteed or endorsed by the publisher.
